# ICT in Nursing and Patient Healthcare Management: Scoping Review and Case Studies

**DOI:** 10.3390/s24103129

**Published:** 2024-05-14

**Authors:** Sara Jayousi, Chiara Barchielli, Marco Alaimo, Stefano Caputo, Marzia Paffetti, Paolo Zoppi, Lorenzo Mucchi

**Affiliations:** 1 ICT Applications Lab, PIN—Polo Universitario “Città di Prato”, 59100 Prato, Italy; 2 Management and Health Laboratory, Institute of Management, Sant’Anna School of Advanced Studies of Pisa, 56127 Pisa, Italy; 3 Department of Nursing and Midwifery, Local Health Unit Toscana Centro, 50134 Florence, Italy; marco.alaimo@uslcentro.toscana.it (M.A.); marzia.paffetti@uslcentro.toscana.it (M.P.); paolo.zoppi@uslcentro.toscana.it (P.Z.); 4 Department of Information Engineering, University of Florence, 50121 Florence, Italy; stefano.caputo@unifi.it (S.C.); lorenzo.mucchi@unifi.it (L.M.)

**Keywords:** Informationand Communication Technologies, healthcare, nursing, patients’ management, Internet of Things, Artificial Intelligence, health monitoring

## Abstract

Over the past few decades, Information and Communication Technologies (ICT) have revolutionized the fields of nursing and patient healthcare management. This scoping review and the accompanying case studies shed light on the extensive scope and impact of ICT in these critical healthcare domains. The scoping review explores the wide array of ICT tools employed in nursing care and patient healthcare management. These tools encompass electronic health records systems, mobile applications, telemedicine solutions, remote monitoring systems, and more. This article underscores how these technologies have enhanced the efficiency, accuracy, and accessibility of clinical information, contributing to improved patient care. ICT revolution has revitalized nursing care and patient management, improving the quality of care and patient satisfaction. This review and the accompanying case studies emphasize the ongoing potential of ICT in the healthcare sector and call for further research to maximize its benefits.

## 1. Introduction

In the contemporary healthcare landscape, the integration of ICT plays a pivotal role in reshaping the responsibilities of nurses. ICT empowers nurses to provide more precise and personalized care by leveraging digital tools and data-driven insights. Younger professionals exhibit a natural inclination towards adopting new technologies, fostering a progressive and tech-savvy healthcare environment. An open and collaborative work environment, where nurses can share concerns and participate in the selection and implementation of ICT, can be instrumental in overcoming resistance from skeptical nurses (or laggars) [1,2,3]. The seamless incorporation of ICT into nursing practices enhances efficiency, accuracy, and ultimately, patient outcomes. Nurses, as architects of patient care, find ICT to be a valuable ally in creating tailored and patient-centric healthcare journeys [4]. The acceptance and effective use of technology are key determinants in navigating the evolving landscape of healthcare services. As the healthcare sector embraces digital transformation, nurses must continually adapt, ensuring they harness the full potential of ICT to meet the dynamic needs of modern healthcare delivery. In essence, the synergy between nurses and ICT is not just a technological integration but a transformative force driving positive changes in healthcare delivery. This article examines how ICT are transforming nursing practice. We highlight a dynamic cycle where nurses’ needs for better care delivery drive the exploration of new ICT tools. These tools, in turn, create new opportunities for nurses, leading to tangible improvements in patient care. In particular, to demonstrate the process of aligning ICT with patient-centered care, we adhered to a logical structure that resulted in the following paper organization: Methods and Materials section (Section 2) reports both a scoping review on the adoption of ICT in nursing care (Section 2.1) and a synthesis of the potentialities of the current and future ICT (Section 2.2); in the Results section (Section 3) the noteworthy aspects derived from the review are described (Section 3.1) and the main identified case studies are considered together with the ICT overview output as an input for mapping the technologies into the health IoT (Internet of Things) architecture layers and their components (Section 3.2); in the Discussion section (Section 4) the presented outcomes are discussed focusing on the Nursing Care Performance Framework (NCPF) [5], taken as a reference to highlight the impact of ICT in nursing system components (Section 4.1), on a quantitative performance assessment (Section 4.2) and on the ICT acceptance by highlighting the benefits of the adoption of such technologies both from patients’ and nurses’ perspectives (Section 4.3). In addition, challenges and barriers to ICT implementation are addressed (Section 4.4). Finally, concluding remarks are reported in Section 5.

## 2. Materials and Methods

A scoping review was performed to offer a broad overview of existing literature on this specific topic and the main achieved results lead to the identification and definition of relevant case studies in this context. The detailed insights provided may help to understand how concepts translate into practice and pinpoint the different contextual factors that may occur. While the synthesis of the existing and future ICT was performed to highlight the technologies potentialities in different case studies that may include: health and life style monitoring, prevention and treatment management, patient’s path management, tele-consultations and data sharing, remote laboratories, learning and training programs, virtual coaching and social inclusion.

### 2.1. Scoping Review: ICT in Nursing and Patient Healthcare Management

To create upright and purposive results the five stages Arksey and O’Malley’s framework [6] was used, as it is widely recognized to be a solid tool to conduct scoping reviews. A brief description of the steps taken is given: (*step 1*) a broad research question was identified (search algorithms), (*step 2*) relevant studies were independently identified (*step 3*) and then assessed by different researchers on the basis of a list of agreement points and applied to the pool of potentially relevant studies retrieved the double abstract and text selection (*step 4*) extraction of the key information from the selected studies and their organization in an explanatory table (*step 5*) summarizing and thematic analysis was conducted and included in the table. The details of the phases are following:

- *Step 1.* To gain a comprehensive understanding of the breadth and depth of the topic, a search was performed in September 2023 using the following algorithm and databases: (ICT OR “Information and Communication Technology”) AND (nurse OR nurses) AND (“patient healthcare management” OR “healthcare management”) Google Scholar, PubMed, Trip Database. This search returned 217 records.
- *Step 2 and 3.* Three researchers independently read both titles and abstracts with the task of including appropriate articles; these latter ones were required to necessarily cover all three elements considered (i.e., ICT, nurses, and patient healthcare management) to highlight studies that could address real-world applications or establish levels of evidence (effectiveness) for guidelines derived from them. Kirk et al. [7] state that research on Digital Nursing Technologies (DNT) are at the centre of significant interest, which leads to the existence of many research directions using a variety of methodologies. While this is positive on one hand, on the other hand, it makes it challenging to compare their effects. After duplicates exclusion, 33 records remained. The researchers subsequently read the full texts and, after a discussion on their relevance, agreed to include 18 records.
- *Step 4 and 5.* Results are charted in Table 1, Table 2, Table 3, Table 4 and Table 5 Key included information is as follows: citation, article type, main results, noteworthy aspects conveyed.

### 2.2. Overview of the Current and Future ICT for Healthcare Services

In recent years, the evolution of technologies in healthcare has revolutionized the management and monitoring of health, revitalized nursing care and patient management by improving the quality of care and patient satisfaction [26,27,28]. ICT has been a key driver of innovation in healthcare for decades [29], and it is likely to continue to play a major role in the years to come. Some of these solutions, such as Electronic Health Records (EHRs), have been in use for many years and have become a standard component of healthcare settings. EHRs have been shown to improve nursing efficiency by reducing the time spent on documentation and providing easy access to patient information. They can also improve patient care quality by reducing medication errors and promoting evidence-based care. Other ICT solutions, such as telehealth and telemedicine, are newer and have only recently gained widespread adoption. However, these technologies have the potential to revolutionize healthcare delivery by providing remote access to care and improving patient outcomes [30]. Existing and future technologies focus on various key areas:

- ***High-precision location-based services for personalized healthcare apps*** [31], fostering overall well-being and care management are some of advancements ICT may introduce in the healthcare context.
- ***6G*** with the enhanced data rates and lower latency compared to 5G enable real-time remote patient monitoring, enabling swift intervention and proactive health management [32,33,34].
- ***Internet of Things (IoT) devices*** and the upcoming ***Internet of nano bio things*** play a pivotal role, gathering real-time health data for remote monitoring and quick intervention. Smart home devices simplify the management of lighting, heating, security, and other aspects of the environment, aiding in monitoring well-being and providing timely support [35,36,37].
- ***Artificial intelligence and machine learning*** lead to intelligent healthcare systems for data analysis and personalized recommendations which may support both patients in the management of their health and professionals in their work activities (e.g., health issues prediction, assistance in monitoring vital signs for a more quick innervation in case of emergencies) [38].
- ***Edge computing, fog computing and cloud computing***, opportunely used depending on the system and specific service requirements, enable real-time processing, allowing smart healthcare systems to remotely detect and address health issues (e.g., effective alert generation for timely decision-making) [39].
- ***Augmented and virtual reality technologies*** facilitate remote medical consultations, therapy and innovative training programs making healthcare more accessible and distributed over the world. Moreover they can also enhance social interactions, provide cognitive stimulation, and contribute to rehabilitation and physical therapy [40].
- ***Assistive robots*** can perform tasks such as delivering medications, reminding individuals to take their medicines, and monitoring changes in behavior or mobility [41].
- ***Drones***, in the future, could be used to deliver medicines or other essential goods directly to patients’ home, reducing the need for in-person visits [42].
- ***Personalized patient interaction technologies*** enhance patient satisfaction and system acceptance. In chronic disease management may improve treatment adherence, and facilitate better health outcomes. However they also play a pivotal role in patients well-being and education, positively affecting their health status.
- ***Innovative interactive user data-driven interfaces***, such as voice-activated virtual assistants can become a personalised health coach which support the users by providing real-time suggestions or reminders based on both the collected data and the user interactions itself. The virtual assistance may also provide information, play music, and control other devices in the home through voice commands, making it easier for patients with mobility problems to interact with their environment [43].
- ***Human-Bond Communication*** (HBC), looking ahead, introduces olfactory, gustatory, and tactile sensations, revolutionizing remote assistance. This holistic approach allows healthcare providers to receive additional sensory information during virtual connections, enhancing their ability to understand the health needs of individuals in a more comprehensive manner [44,45].

## 3. Results

This section reports the results of the scoping review and the ICT mapping into the healthcare service oriented architecture layers and components. These elements represent the basis of the discussion provided in Section 4. It is worth highlighting that the main results of the scoping review lead to the identification and definition of relevant case studies in this context, which range from health and life style monitoring to treatment management and virtual coaching, but also patient management visit scheduling, virtual consultations, remote laboratories, learning and training programs, etc. These are considered as an input for the mapping of the ICT into the health IoT architecture.

### 3.1. Scoping Review Results: ICT in Nursing Care

This section presents the key findings from our systematic analysis of existing research on ICT in nursing care. It’s not an in-depth analysis of specific studies, but rather a broad overview of the current landscape in this field.

As aforementioned, 18 articles were retrieved. Two case studies, an Randomized Controlled Trial (RCT) protocol, a framework study, two among report and editorial, three studies utilizing surveys and other qualitative techniques, and eight among comparative, integrative, and systematic reviews were included.

Ayanlade et al. [8] conducted a survey among two different groups of people, namely nurses and patients, to gather primary data on their opinions and perceptions towards Heath Information Technology (HIT) in the management of a chronic condition, type I diabetes. A general positive attitude towards HIT is evident, but there’s also a significant divergence in terms of the perceived risks it may pose. Nurses express concerns about potential job loss due to “technological replacement”, while patients express fear of possible data security breaches. It immediately becomes clear that different stakeholders understand that the potential improvements that HIT will bring are not devoid of negative aspects, which will need to be managed from an organizational standpoint. Gund et al. [9] also find a positive attitude of nurses towards ICT, even if a different attitude is found in the physician group of the attitude survey. There is evident lower confidence in the development of home-based patient follow-up practices, which the authors attribute to different working routines. Recent literature on Hospitalization at Home (HaH) depicts it [46] as a reality which has proven to be “more efficient and effective than conventional hospitalization”, but it requires a clinically sound team to manage it. It may be argued that only mature organizations, those who embrace e-transformation and include e-health and ICT as staff’s core competencies can make the most of all the possibilities that ICT offers [47]. Giordano et al. [21] illustrate the possibility of using ICT solutions for aspects such as fall prevention, with the use of tele-management programme, which configures a “technological-care integration” as an active tool [48]. Contrary to what is asserted, Buyl et al. [11], in their systematic review, assert that little evidence is available, thus it is appropriate to proceed with caution in assessing the performance of these new solutions.

The selected studies tend to emphasize the need for further exploration in the realm of healthcare and ICT integration, and they tend to be divided between highlighting the sometimes positive, sometimes negative aspects that this integration either generates or fails to generate. On the positive side included studies underlined the role of ICT solutions in supporting clinical and team decisions [15,16,18,22,23] equally emphasizing the importance of a strong governmental commitment [20] as well as authorities’ pledge to investment in professionals’ and patients’ training [24] and an in line development of practices with quality guidelines [14], while not forgetting to formalize internal governance structures for the implementation process of the new solutions [12]. Although mixed evidence on the possibilities of an “information improved health” [13] exist, authors are willing to pushing forward with the generation of new evidences to correctly report it to the scientific community. Wildevuur et al. [19] particularly emphasize the need to move in this direction through pathways of co-designing and co-management between patients and professionals. Cantor and colleagues [17] express the existence of limited evidence of effectiveness on interventions such as telehealth replacing in-person meetings in specific sectors, such as contraceptive care and IPV services.

From a specifically nursing perspective, the systematic review by Rouleau and colleagues [29] highlights how various dimensions of work are positively influenced by incorporating ICT-related solutions into the processes of nursing care delivery. This work is particularly interesting because it uses the NCPF [5] to prove that ICT solutions have a direct impact on 19 indicators (i.e., “documentation time, time spent for patient care, time management, knowledge updating and utilization, information quality and access, nurse autonomy, intra and interprofessional collaboration, nurses competencies-skills, nurse-patient relationship, quality of documentation, assessment, care planning and evaluation, teaching of patients and families, communication and care coordination, nurses’ perspectives of the quality of care provided, patient comfort and quality of life related to care, empowerment, functional status, and satisfaction or dissatisfaction of nurses and patients using ICT”).

This last aspect will be further developed in the discussion section, serving as one of the focal points of this exposition. ICT has very real effects on care processes, enhancing them from various perspectives. The proposed framework (NCPF) will serve to systematize the multiple points of intersection between the nursing dimension and the technological dimension, highlighting how these points of contact give rise to tangible and measurable benefits in practice, ultimately benefiting the patient.

A particular contribution is provided by Chien et al. [10] as it highlights the phenomenon of BYOD and its expansion into the healthcare field, pointing out its potential of reducing clinical burden and providing practical solution for nurses where ICT solutions are not in place.

While a scoping review was performed to offer a broad overview of existing literature on this specific topic, a case study was included to allow a specific real-life scenario. The detailed insights provided may help to understand how concepts translate into practice and pinpoint the different contextual factors that may occur.

### 3.2. Mapping ICT into a Healthcare Service Oriented Architecture

To better understand which and how current and future ICT can support nursing and patient healthcare management system, the usage of ICT in Nursing and Patient Healthcare are mapped into an IoT Healthcare Service Oriented Architecture. The main aim is to provide the reader with a structured framework for integrating current and future ICT solutions within healthcare systems.

**Figure 1:**
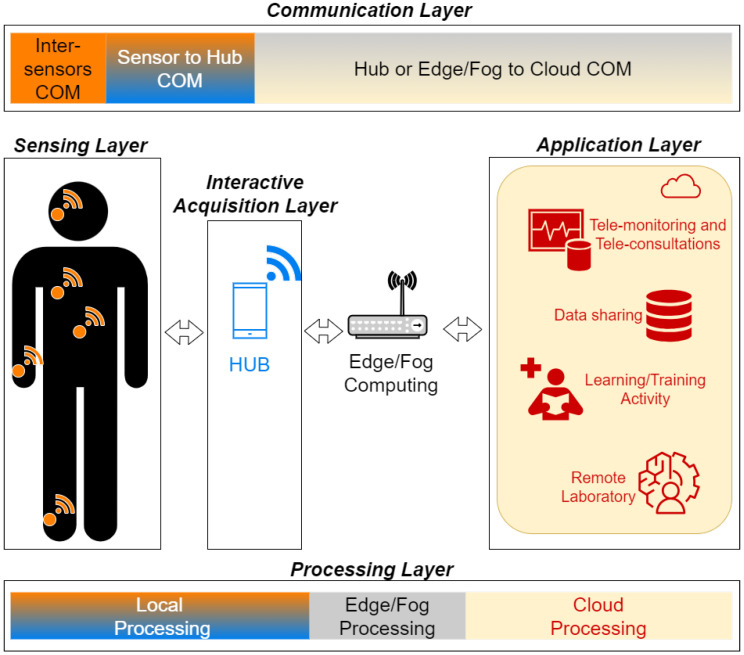
Health IoT Architecture.

In Table 6 and Table 7 the main ICT are mapped into a 5-layer reference Health IoT Architecture (Figure 1):

- *Sensing Layer.* It enables the collection of data directly from sensors ranging from health and activity parameters monitoring devices to behavior and environmental monitoring systems. They include in-body, on-body and off body sensors, but also environmental and bio-metric IoT sensors.
- *Interactive Acquisition Layer*. It allows data gathering through the use of real-time user input and feedback interactive interfaces (e.g., smart adaptive virtual coach or assistance).
- *Communication Layer*. It enables data exchange among the different network nodes of the architecture (e.g., sensors, hub, cloud).
- *Processing Layer*: It is responsible for analysing and processing data closer to the source (edge computing), in cloud (cloud computing) or in between them (fog computing) based on the system requirements and available resources.
- *Application Layer*: It is in charge of providing applications and interfaces that leverage the insights derived from the collected data (e.g., dashboards, analytics tools, monitoring platforms, etc.).

**Table 6:** Mapping ICT into IoT Architecture Layers (From Sensing to Communication Layer).

Architecture Layer	Component	Technology
Sensing	Health parameters monitoring [45,49,50,51]	- In-body, on-body and off body sensors for real time health data acquisition and tracking (e.g., heart rate, heart rate variability, SpO2, ECG, photoplethysmogram, implants, nanoscale and biological devices, etc.) - Internet of things and Internet of nano bio-things - Senducers (Human Bond Communication)
	Activity monitoring [52,53,54]	- IoT cameras, motion tracking sensors and presence sensors for tracking daily activities, gait pattern, tremor, fall detection, position, step count, distance traveled, exercises etc.
	Behavior monitoring [55,56]	- Wearable, environmental, bio-metric IoT sensors for tracking sleep patterns, social interactions, stress levels, gesture recognition, home space utilization, location etc. - Online and social media activity monitoring for tracking patient interactions, content consumption, and online preferences.
	Environmental monitoring [57]	- Temperature, air quality, humidity, and allergen levels sensors for monitoring the environmental factors that may affect the patient’s health status.
Interactive Acquisition	Lifestyle monitoring [58]	- Real-time user input and feedback Interfaces (e.g., text, voice, gesture, touch, bio-metric based interfaces, eye tracking interfaces, etc.)
	Therapy Adherence [59,60,61]	- Alert, reminder and notifications mechanisms that include user feedback
	Needs Tracking [43,62,63]	- Virtual Coaching and Assistence - Adaptive and customized interactive interfaces based on specific users’ real time needs
	System Acceptance [64,65,66,67]	- System usability tracking (user-transparent data flow monitoring, support and assistance requests tracking, training and learning contents usage monitoring, virtual assistant, etc.)
Secure Communication [68,69,70,71,72,73,74]	Inter-sensors communication (in- and on-body)	- Biological Communication (Molecular Com) - Classical Communication (e.g., acoustic, nano mechanical, electromagnetic, etc.) - Electromagnetic, optical ultrasound, conductive body proprieties
	Sensors to hub communication	- Bluetooth (BLE), NFC, IEEE 802.15.6, IEEE 802.15.4, G.9959, Zigbee, SmartBAN, RFID, etc. - Human Bond communication
	Hub to cloud communication and vice versa	- Low Power Wide Area Network (LPWAN), SigFox, WiFi, Low Power Wide Area Network (LoRAWAN), IEEE 802.16, LTE, 4G/5G, etc.

**Table 7:** Mapping ICT into IoT Architecture Layers (From Processing to Application Layer).

Architecture Layer	Component	Technology
Processing (oriented to Application)	Data analysis and classification [75,76,77,78]	- Data mining, correlation and regression analysis, time-based and cluster analysis - Machine Learning and AI-Based classification - Expert-driven classification tuning based on additional data (e.g., metadata tagging)
	Data processing and correlation [79,80,81,82,83,84]	- Edge, Fog and Cluod Computing - Machine Learning and Artificial intelligence - Behavioral Analytics and gesture recognition - Predictive Analytics
	Alert generation for Decision Making support [85,86,87,88]	- Data and events correlation, anomaly detection, parameter threshold-based trigger, rule-based alerts. - Dynamic decision support systems
	Customized content generation for a personalized patient interaction [89,90,91,92]	- Natural Language Processing technology - Application- oriented content creation (static and interactive) based on the data analysis (e.g., treatment plan, visit scheduling, lifestyle advice, prevention and health management advice, training etc.)
Application	Tele-monitoring (nurse) [93,94]	- Patient’s healthcare status progress monitoring innovative multimedia interfaces
	Treatment Management (nurse) [95,96]	- Exploiting AI algorithms for dynamic updates based on the patient’s healthcare status progress, adaptive and real-time therapy reminders.
	Visit Planning management [97,98]	- Exploiting data analysis for time and resources management, patient visit planning.
	Data sharing and communication (patient/nurse) [99,100,101,102]	- Efficient data sharing interfaces - Automatic short report creation - Real-time translation tools for overcoming linguistic barriers.
	Tele-consultations (patient-nurse, nurse-nurse, etc.) [103,104,105,106]	- Video conference and data sharing tools - Augmented Reality for advanced interactions among professionals
	Remote Laboratory (nurse) [107]	- Video conference and data sharing tools, avatar, AI, Augmented Reality, Virtual Reality for remote guiding.
	Learning/training (patient/nurse) [108,109,110]	- Virtual coaching - Augmented Reality educational and training programs for both patients and nurses.
	User Acceptance management (patient/nurse) [111,112,113]	- Tools for gathering end-user feedback, such as system usability scale (SUS), user experience questionnaire (UEQ), technology acceptance model (TAM), Unified Theory of Acceptance and Use of Technology (UTAUT) and customized questionnaire defined based on the specific system under evaluation. - Advanced user-transparent monitoring mechanisms for gathering information on the use of the system by the users’ and dynamically help them in real time (e.g., support of an adaptive virtual assistant).

Focusing on the nursing and patient healthcare management, for each layer, different components are identified and the main suitable technologies are listed.

## 4. Discussion

The outcomes presented pave the way for the examination of two primary subjects: ICT in NCPF and ICT acceptance. Both of them are addressed in the following subsections.

### 4.1. ICT in Nursing Care Performance Framework

Starting from the theoretical foundations for the NCPF [5], and the concept of a nursing system as an open system composed of interrelated subsystems, the main objective of this section is to highlight the role of ICT in supporting the system in acquiring inputs from its environment, participating in transformation processes, and generating output that contributes additional value to its context. To achieve this goal the three NCPF functions are analysed and for each of them the tasks the adoption of ICT may enhance are presented. The three functions can be seen as three case studies where the ICT can be implemented for improving the performance indicators of a nursing system: (i) acquiring, deploying, and maintaining nursing resources (ii) transforming nursing resources into nursing services (iii) producing changes in a patient’s condition as a result of providing nursing services.

#### 4.1.1. Function 1: Acquiring, Deploying and Maintaining Nursing Resources

This function includes all the characteristics that affect the ability of the nursing system to meet healthcare needs, ranging from the dynamic capacity to acquire and maintain the necessary resources to develop new resources or improve their allocation.

Providing efficient nursing services relies on having a staff with the requisite skills and competencies to address patients’ specific needs promptly, which include also managing new facilities such as multiple electronic devices and applications due to the progress of digitalization [29].

The adoption of ICT may help in:

- delivering advanced training programs that can dynamically adapt to the skill and experience level of the nurses, besides supporting them in real-time in their tutoring activity towards their patients;
- improving working conditions thanks to new facilities (e.g., tools for patient data monitoring, for an easy interaction between patients even in case of cultural and linguistic barriers, for real-time data sharing among colleagues aimed at achieving a better coordination or asking for support in difficult conditions, for assistance planning, etc.);
- efficiently allocating resources based on patient conditions, which may include prediction algorithms for planning support;
- reducing costs due to improvements in resources management and staff coordination, besides data gathering and analysis for future planning.

#### 4.1.2. Function 2: Transforming Nursing Resources into Nursing Services

This function represents the activities performed by nurses to convert the available resources into nursing services that meet patients’ needs. This includes the nursing system’s capacity to coordinate the efforts and ensure the seamless operation of processes related to services delivering. Also, how patients actively participate in their care processes and how both nurses and patients navigate their care experiences are encompassed together with the creation and maintenance of values and standards to be followed in the design of nursing services [114,115].

The adoption of ICT may help in:

- simplifying process such assessment, planning, evaluation, problems and symptoms management;
- providing high personalized services based on real-time patients’ collected data opportunely processed for enhancing and supporting coordinated decision making processes, defining and updating patients’ path and visits time cards;
- defining prevention programs;
- enhancing care coordination among professionals for an efficient nursing care delivery process.

#### 4.1.3. Function 3: Producing Changes in a Patient’s Condition as a Result of Providing Nursing Services

This function refers to the nursing system’s ability to accomplish its mission and foster a positive state in its interactions with its surroundings. Dynamic interactions among patients, nursing staff, and nursing procedures aim to contribute to favorable alterations in a patient’s functional status, disease condition, or evolving situation [116].

The adoption of ICT may help in:

- integrating care, identifying risks, preventing errors and adverse events both by tele-monitoring system and behaviour analysis;
- enhancing patient quality of life by meeting patients’ care needs, including nutrition, psycho-physical symptom management and avoiding unnecessary interventions;
- increasing patients’ knowledge, skills and improving their awareness of self-care through the delivery of health-promoting behaviors (e.g., understanding prescribed treatments, recognizing symptoms, etc.);
- contributing to the improvement of a variety of elements related to patients’ overall functional well-being, covering physical, psycho-social, and cognitive aspects, depending on their specific needs;
- tuning the provision of care services and models based on patients’ satisfaction with their care experience and the evolution of their condition.

### 4.2. Quantitative Performance Assessment

While our paper focused on the potential of ICT to improve communication and collaboration in nursing care, we acknowledge the lack of specific data on performance and patient outcomes. Papers in literature address the topic, but the documents do not provide specific quantitative results on the impact of ICTs on nursing care performance. However, several papers outline the use of the Nursing Care Performance Framework (NCPF) to analyze the influence of eHealth domains on nursing care, including management, computerized decision support systems, communication, and information systems. The primary outcomes studied included nurses’ practice environment, nursing processes, professional satisfaction, and nursing-sensitive outcomes. The secondary outcomes considered satisfaction or dissatisfaction with ICTs from both nurses’ and patients’ perspectives [29,117].

In [118], the authors claim to give a sort of quantitative evaluation, but based on questionnaires, not on field experimentation. The results from the study on the impact of ICT on nursing care performance revealed that ICT, as a component of knowledge management infrastructure, positively and significantly influences knowledge management processes in the selected teaching hospitals. Specifically, the findings indicated that information technology, along with organisational structure and organisational culture, plays a crucial role in enhancing knowledge acquisition, conversion, application, and protection within the nursing care environment. These results suggest that the effective use of ICT in knowledge management processes can contribute to improving nursing care performance in healthcare organizations. By leveraging ICT tools and infrastructure, nurses can enhance their decision-making processes, communication, and overall efficiency in delivering quality patient care.

### 4.3. ICT Acceptance

As highlighted by the selected and reviewed articles, the adoption of ICT in the considered context needs to be analysed following a multidisciplinary approach, which includes the different end-users’ perspectives. Both patients and nurses’ acceptance is essential for the adoption of a new technological systems in their lives and work activities, respectively. Starting from the information gathered in Table 6 and Table 7, in the following the potentialities of ICT are briefly described for each end-user.

#### 4.3.1. User Acceptance: Patients Side

In the Health IoT Architecture, a patient represents both a source and a destination of information. He can be seen as a node of the network able to provide data which are the basis for the functioning of any kind of healthcare applications. However, he is also the destination of some information processed by the system or directly coming from the healthcare professionals. Being a source of data rises many issues related to the privacy aspects (which are beyond the scope of this contribution), on the other side the patient may benefit from collecting and sharing his data (e.g., health parameters, daily activities, etc.) with professionals both for self-monitoring and health management. The processing of lifestyle, health and disease monitoring data together with additional data coming from other sources leads to essential information destined to patients for different purposes ranging from healthcare and treatment management (e.g., reminder, dynamic updates) to care routine and time management (e.g., virtual coach). This enables patient empowerment and awareness in the management of his personal health and enhance the communication and coordination between him and those involved in his healthcare management.

While the Health IoT architecture offers clear benefits, user acceptance hinges on understanding both the potential and the anxieties surrounding data ownership and utilization. Studies have shown that patients are generally receptive to ICT in healthcare if they perceive a clear gain in terms of self-management, improved communication with healthcare providers, and a sense of control over their health data [119]. However, concerns regarding data privacy and security remain a significant barrier to adoption [120]. Addressing these concerns through transparent communication, robust security measures, and user-friendly interfaces will be crucial for fostering trust and promoting long-term user engagement with Health IoT technologies.

#### 4.3.2. User Acceptance: Nurses Side

As for a patient, in the Health IoT Architecture, a nurse represents both a source and a destination of information. Also, he can be seen as a node of the network in charge of providing data input for decision making based on real-time patients’ data gathered and processed by the system (e.g., therapy updates, dynamic assistance planning, training programs, etc.).

In a recent study on nurses working in technology-intensive contexts, such as Intensive Care Units or Robotic Surgery Operation Theatres, an investigation of the determinants of acceptance is provided [121]. Peer influence and acceptance influences nurses’ behaviour towards new technologies, the perception of being capable of using ICT is determining the effective use of technologies and the evidence the nurses get from providing an enhanced quality assistance is a pivotal factor in their acceptance and effective use of technologies. Of course, the age of the professional is a determining factor. Young nursing professionals are naturally less hesitant or resistant to the inclusion of new technologies in their work routine. However, it should be emphasized that a collaborative work environment also influences even the more naturally skeptical professionals towards the acceptance and use of ICT.

Traditionally seen as separate domains, technology and nursing care are now harmoniously intertwined, as evidenced by Locsin’s theory [122]. Locsin argues that ICT are not meant to replace nurses, but rather to augment their ability to care for patients “as a whole” [123]. A nurse’s core competency lies in continuous patient observation. ICT empowers nurses to perform this observation with greater depth and immediacy. By providing nurses with precise data and streamlined workflows, ICT enables them to deliver more effective and personalized care. This translates to care pathways that are increasingly tailored to individual patient preferences. Ultimately, this technology-driven approach transforms patients from passive recipients of care into active participants in shaping their own healthcare journey.

### 4.4. Challenges and Barriers to ICT Implementation

In addition to highlighting the benefits of ICT integration in nursing and patient healthcare management, it is imperative to acknowledge and address the multifaceted challenges and barriers inherent in its implementation.

Embracing ICT in healthcare often requires navigating intricate technical landscapes. Issues such as interoperability among different systems, data security, and infrastructure limitations pose significant challenges. Interoperability gaps hinder the seamless exchange of patient information between disparate systems, potentially compromising patient care continuity. Moreover, ensuring robust cybersecurity measures is paramount to safeguard sensitive patient data from breaches and cyber threats. Addressing these technical challenges demands concerted efforts in standardization, investment in robust IT infrastructure, and ongoing technological advancements.

The integration of ICT in healthcare raises complex ethical dilemmas that necessitate careful consideration. Maintaining patient privacy and confidentiality amidst the digitization of health records remains a paramount concern. Striking a delicate balance between leveraging patient data for improved care outcomes and respecting individual autonomy and privacy rights is imperative. Ethical frameworks and guidelines must be rigorously upheld to mitigate risks of data misuse and breaches of trust. Moreover, ensuring equitable access to ICT-enabled healthcare services is essential to prevent exacerbating existing disparities in healthcare delivery.

Organizational culture and dynamics play a pivotal role in shaping the successful implementation of ICT in healthcare settings. Resistance to change, insufficient training programs, and workflow disruptions pose formidable barriers. Cultivating a culture of innovation and fostering interdisciplinary collaboration are pivotal in overcoming organizational inertia. Adequate training and support programs should be instituted to empower healthcare professionals with the requisite digital literacy and skills to harness ICT tools effectively. Furthermore, streamlining workflows and ensuring seamless integration of ICT into existing practices are essential for optimizing operational efficiency and enhancing patient outcomes.

Technical complexities form the foundation upon which successful ICT integration rests. One major hurdle is interoperability. Imagine a nurse piecing together a patient’s medical history from fragmented records because different healthcare systems don’t “talk” to each other. This lack of seamless data exchange hinders care coordination and can compromise patient safety. Another technical complexity lies in ensuring robust data security. EHRs are prime targets for cyberattacks, demanding robust encryption and access controls to safeguard sensitive patient information. Finally, inadequate infrastructure, such as outdated hardware, limited bandwidth, or unreliable internet connectivity, can significantly impede the smooth operation of ICT systems. Nurses may experience delays in accessing patient data or encounter system crashes due to these limitations.

Ethical complexities weave intricately with the technical considerations. Maintaining patient privacy is paramount in the digital age. Nurses must navigate the delicate balance between leveraging patient data to improve care outcomes and respecting individual autonomy and privacy rights. Clear guidelines on data sharing and patient consent are crucial to ensure ethical use of patient information. However, the ethical considerations extend beyond individual records. The increasing use of AI-powered healthcare tools introduces the risk of perpetuating existing biases in healthcare delivery. Nurses need to be aware of these biases and how they might impact patient care decisions. Additionally, ensuring equitable access to ICT-enabled healthcare services is essential. Not all patients have equal access to technology or the digital literacy skills needed to navigate these systems. Nurses play a vital role in ensuring inclusive healthcare delivery and bridging the digital divide for patients with limited access.

Organizational complexities further complicate the implementation process. Resistance to change can be a significant barrier, with some healthcare professionals hesitant to adopt new technologies due to fear of increased workload or disruption to established workflows. Cultivating a culture of innovation and fostering interdisciplinary collaboration are essential to overcome this resistance. Furthermore, adequate training and support programs are crucial to empower nurses with the requisite digital literacy and skills to effectively utilize ICT tools. Lack of ongoing support can lead to frustration and hinder user adoption. Finally, simply introducing new technology isn’t enough. ICT systems need to be seamlessly integrated into existing workflows to optimize efficiency and minimize disruption to patient care routines.

## 5. Conclusions

This study presents the significant impact of ICT on nursing care and patient healthcare management. We have showcased the diverse array of ICT tools used in these crucial healthcare domains, including electronic health records systems, mobile applications, telemedicine solutions, and remote monitoring systems. Our findings highlight how these technologies can transform healthcare delivery by improving the efficiency, accuracy, and accessibility of clinical information, ultimately leading to better patient care outcomes. The adoption of ICT has rejuvenated nursing care practices and patient management processes, resulting in higher quality care and increased patient satisfaction. Nevertheless, our study also acknowledges the challenges and opportunities associated with ICT in healthcare. Further research and development efforts are needed to fully realize the benefits of ICT applications, particularly in terms of interoperability, data security, and user acceptance. Looking ahead, it is imperative for healthcare systems to continue investing in ICT infrastructure and initiatives that prioritize patient-centered care principles. By effectively leveraging ICT, healthcare providers can optimize their services, enhance healthcare delivery, and improve patient outcomes.

## Figures and Tables

**Table 1:** Selected Surveys.

Article	Main Results	Theme and Noteworthy Aspects Conveyed
[8]	Staff: perception of Health Information Technology (HIT) as an help for their job, but fear job loss. Patients: fear data insecurity.	Diabetes care and management. Ease of use is paramount to staff.
[9]	Healthcare professionals: positive to both current and future use of ICT tools in healthcare and home follow-up.	Physicians’ relatively lower enthusiasm for home follow-up compared to nurses may be attributed to variations in their working routines.
[10]	Improving nursing staff satisfaction with Bring Your Own Device (BYOD) systems involves adding practical features and reducing the associated clinical burden.	BYOD enables professionals to utilize their personal devices for work-related activities, such as accessing patient records and carrying out job-associated tasks.

**Table 2:** Systematic Reviews.

Article	Main Results	Theme and Noteworthy Aspects Conveyed
[5]	Extraction tool: NCPF	ICT have diverse effects on 19 nursing care indicators, encompassing documentation time, patient care, time management, knowledge utilization, information quality, nurse autonomy, collaboration, competencies, nurse-patient relationships, documentation quality, assessment, care planning, patient education, communication, care coordination, perspectives on care quality, patient comfort, empowerment, functional status, and satisfaction.
[11]	Limited evidence found on supporting the effectiveness of e-Health interventions	E-Health interventions have the potential to enhance physical activity, promote healthy behaviors, yield positive psychological outcomes, and have favorable effects on clinical parameters.
[12]	Research focuses on technology-supported interventions aimed at alleviating loneliness and social isolation among older adults experiencing reduced mobility.	All interventions yielded positive results, indicating their feasibility. Notably, desktops/laptops constituted a significant portion of the devices utilized for support. Furthermore, most interventions facilitated interaction within online groups rather than one-on-one arrangements tailored for the intervention.
[13]	A comprehensive review of automated-entry Patient-Generated Health Data (PGHD) devices and mobile apps for the prevention or treatment of 11 chronic conditions.	In general, PGHD devices offer abundant information to both patients and providers. However, the extent to which this information has demonstrably enhanced health outcomes remains uncertain, with mixed evidence in this area.
[14]	The results highlight the importance of leveraging information technology to enhance early warning and clinical handover systems in healthcare settings for improved patient outcomes and resource utilization.	Development of electronic early warning and clinical handover systems should align with established guidelines.
[15]	The strategic planning of the organization serves as a crucial factor that moderates the relationship between the other independent variables and the dependent variable.	By having direct access to data, information, and knowledge related to health issues, and with information tailored around the patient, healthcare providers can share and access a broader range of patient data, allowing for more focused attention and ultimately increasing the benefits for the patients.

**Table 3:** Selected Reviews.

Article	Main Results	Theme and Noteworthy Aspects Conveyed
[16]	This integrative review takes into consideration 14 new studies, focusing on the time range 2009–2019.	ICT brings benefits such as the control of non-communicable diseases, education, and health promotion, serving as potential avenues for overcoming inequalities in healthcare.
[17]	Assess the efficacy, utilization, and implementation of telehealth for women’s preventive services in reproductive healthcare and Inter-Personal Violence (IPV). Also, examine patient preferences and engagement with telehealth, particularly in the context of the COVID-19 pandemic.	Limited evidence suggests that telehealth interventions for contraceptive care and IPV services result in equivalent clinical and patient-reported outcomes as in-person care.

**Table 4:** Selected Studies.

Article	Main Results	Theme and Noteworthy Aspects Conveyed
[18]	ICT supports the advanced practice: - availability and completeness of patient data - enhanced quality of decision-making - patients path’s appropriateness	- Disruptive vs. sustainable innovations - Higher quality of decision making and improved patient safety
[19]	ICT has introduced novel data-sharing activities and created a new role for data professionals in care provision. Additionally, it has contributed to a carefree lifestyle through the semi-automated management facilitated by the device.	ICT can lead to an experience of partnership between patients and healthcare professionals.

**Table 5:** Other Selected Articles.

Article	Main Results	Theme and Noteworthy Aspects Conveyed
[20]	Nearly 90% of Spain’s general practitioners, pediatricians, and primary care nurses utilize Electronic Health Record (EHR) systems. Moreover, electronic prescription systems are employed in over 40% of Spanish primary care centers and 42% of pharmacies.	Spain’s health services: incorporation of ICT into patient care practices.
[21]	Effects of ICT in a home fall prevention program	Home tele-management programme
[22]	International, a not for profit registered charity, primary care provider	Introducing telemedicine to homes in remote communities eliminates the necessity and challenges of patient travel. This approach is referred to as the Novel Hybrid System of Telemedicine (NHST).
[23]	The medication management process has been thoroughly studied; however, upon closer examination of the literature, the evidence varies across different phases of medication management, groups of individuals involved, and types of MMIT.	Clinical Decision Support Systems (CDSS) and Computerized Provider Order Entry (CPOE) systems have been extensively studied, surpassing other applications of MMIT. Additionally, non-physician groups exhibit distinct preferences, varied needs, and diverse usage patterns in their interactions with MMIT systems.
[24]	Complete training model for resources allocation	Hospitalization at Home (HaH) has demonstrated greater efficiency and effectiveness compared to traditional methods; however, it necessitates a higher allocation of resources and specialized personnel.
[25]	It provides key research areas, evidence-based recommendations, and guidelines to enhance the implementation and adoption of eHealth solutions in healthcare settings.	At guidelines level, which is the highest evidence-based source, we find the strategic importance of eHealth solutions: “Healthcare organizations will ensure continuous executive sponsorship and establish a formal governance structure led by executive leadership to guide the implementation of the eHealth solution”.

## Data Availability

Not applicable.
